# The Association between the Serum Uric Acid Level and Hypertension in Middle-Aged and Elderly Adults

**DOI:** 10.1155/2021/4626062

**Published:** 2021-10-26

**Authors:** Xianpeng Xu, Jinke Huang, Simin Wu, Qingjie Ji, Xuguang Guo, Yong Huang

**Affiliations:** ^1^Department of Acupuncture and Moxibustion, Quzhou Hospital of Traditional Chinese Medicine, Quzhou, China; ^2^The Second Clinical Medical School, Guangzhou University of Chinese Medicine, Guangzhou, China; ^3^Department of Clinical Laboratory Medicine, The Third Affiliated Hospital of Guangzhou Medical University, Guangzhou 510150, China; ^4^School of Traditional Chinese Medicine, Southern Medical University, Guangzhou, China; ^5^Department of Acupuncture and Moxibustion, Nanfang Hospital, Southern Medical University, Guangzhou, China

## Abstract

**Background:**

Studies on serum uric acid (sUA) levels and hypertension (HTN) are controversial. To investigate the association between the sUA level and the incident of HTN in middle-aged and elderly adults, we performed this study.

**Methods:**

6399 participants aged ≥40 years from the National Health and Nutrition Examination Survey (NHANES) were included. Weighted multiple logistic regression analysis was carried out to evaluate the relationship between the sUA level and the incident of HTN, exploring the potential nonlinear relationship using the fitted smoothing curves. If nonlinearity was observed, the inflection point was further calculated by a recursive algorithm.

**Results:**

A positive relationship between the sUA level and the incident of HTN was found. However, it may differ in different race groups, nor between male and female. Moreover, the association between the sUA level and the incident of HTN followed a U-shaped curve in male (turning point: sUA 4.1 mg/dL) and Whites (turning point: sUA 7.9 mg/dL).

**Conclusions:**

The results revealed that the sUA level is positively correlated with the incident of HTN, in middle-aged and elderly adults. However, it followed a U-shaped curve in males and Whites.

## 1. Introduction

Worldwide, hypertension (HTN) is both a disease and a major modifiable risk factor for all-cause morbidity and mortality [1]. It is reported that more than 100 million people are currently affected by HTN and it is expected that more than 29.2% of adults will suffer from this disease in 2025 [2]. HTN rarely causes symptoms in its early stages, so less than half of HTN patients value their condition, although early diagnosis and treatment of HTN are essential [1]. Hence, a better understanding of modifiable risk factors for HTN is useful for early detection and prevention of HTN, which could help reduce this disease and its associated complications.

As a natural component of blood, serum uric acid (sUA) is the ultimate product of purine metabolism [3]. In recent years, epidemiological data have found inconsistent conflicting results on the association between sUA and HTN [4]. Elevated sUA reduces susceptibility to nitric oxide, endothelial dysfunction, and damage to the renal angiotensin aldosterone system, which eventually causes blood vessels to constrict [5, 6]. However, there are many different views on this issue due to the complex factors associated with sUA and HTN and controversial findings have been reported. Specifically, while an elevated sUA level was associated with an increased risk of developing HTN [4, 7–11], most other observational or Mendelian randomized studies did not support these associations [12–14]. Therefore, the causal relationship between sUA levels and HTN remains to be examined. To evaluate the association between them in middle-aged and elderly adults, data from the United States (US) National Health Nutrition and Examination Survey (NHANES) were used to perform this study.

## 2. Methods

### 2.1. Study Population

**Figure 1:**
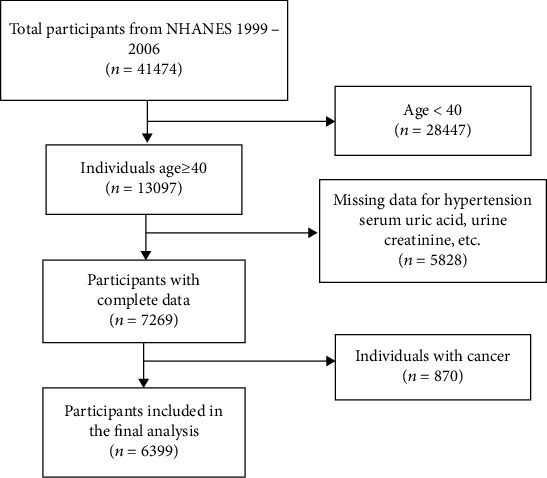
Sample screening flow chart.

The NHANES database collected health examination data from the noninstitutionalized US population [15]. The NHANES study was approved by the Institutional Review Board of the National Center for Health Statistics. 41474 participants were identified from NHANES 2001–2006, and 35075 participants without complete measurement data or the health status did not meet the inclusion criteria were excluded. Finally, 6399 participants with completed data were analyzed in this study (Figure 1).

### 2.2. Study Variables

The exposure variable was serum sUA. From 1999 to 2001, the sUA levels were measured using Roche Hitachi Model 917 or 704 Multichannel Analyzer, while the Beckman Synchron LX20 was used since 2002 [16].

The outcome of interest was blood pressure status measuring by trained research physicians. HTN was defined as self-reported HTN. Subjects with systolic BP ≥ 130 mmHg or diastolic BP ≥ 80 mmHg were considered to have HTN [17].

Additionally, other covariates included age, sex, race, income-poverty ratio, educational level, body mass index (BMI), diabetes mellitus status, physical activity, smoking behavior, alcohol consumption, total cholesterol, serum homocysteine (Hcy), urine creatinine, blood urea nitrogen, and serum calcium.

### 2.3. Statistical Analysis

R (version 3.4.3) and EmpowerStats (X&Y Solutions, Boston, MA) were applied to performed statistical analysis. Sample weights were calculated considering all estimates from NHANES. Categorical variables were presented using frequencies or percentages, and continuous variables were mean ± standard deviation. After adjustment for potential confounders, weighted multivariate linear regression models and smooth curve fitting were performed to evaluate the association of sUA levels with HTN incidence. The weighted linear regression model was performed to calculate the difference of continuous variables; for categorical variables, the weighted chi-square test was used. *P* < 0.05 was considered statistically significant.

## 3. Results

### 3.1. Characteristics of Participants

Characteristics of the included subjects were subdivided followed by the sUA quartiles (Q1: 1.5–4.3 mg/dL; Q2: 4.4–5.2 mg/dL; Q3: 5.3–6.3 mg/dL; and Q4: 6.4–13.7 mg/dL), and the medians of these chosen quartiles were 3.8 mg/dL, 4.8 mg/dL, 5.8 mg/dL, and 7.2 mg/dL. Among different groups of sUA, significant differences in baseline characteristics were observed, with the exception of education, income, physical activity, and total cholesterol (Table 1).

### 3.2. Association between sUA and HTN

**Figure 2:**
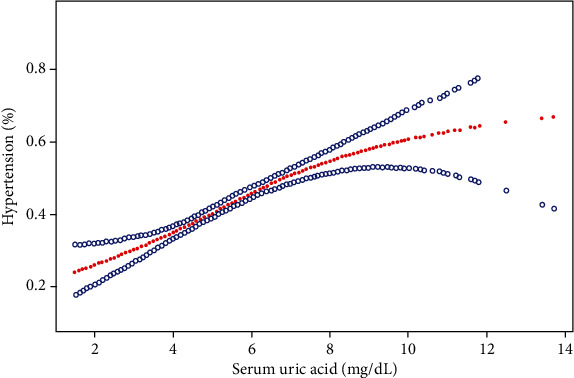
Association between sUA and HTN. The area between two blue dotted lines is expressed as a 95% CI. Age, sex, race, income-poverty ratio, physical activity, smoking behavior, alcohol consumption, diabetes mellitus status, body mass index, total cholesterol, serum homocysteine, urine creatinine, blood urea nitrogen, and total calcium were adjusted.

Three models were developed: model 1, unadjusted; model 2, sex, age, and race were adjusted; and model 3, adjusted for covariates presented in Table 1. In all models, sUA was positively associated to the incidence of HTN (Table 2, Figure 2). After converting sUA from a continuous variable to a categorical variable (quartiles), the odds ratios (ORs) for the association of sUA and HTN in the other three groups were 1.25, 1.53, and 2.11, respectively, with the lowest quartile as the reference. When stratified by sex, a positive association was found between sUA and HTN. When stratified by race, sUA was positively related to the incidence of HTN in Blacks, Whites, and Mexican American, but not in other races (OR 1.14 (0.97, 1.33)).

**Figure 3:**
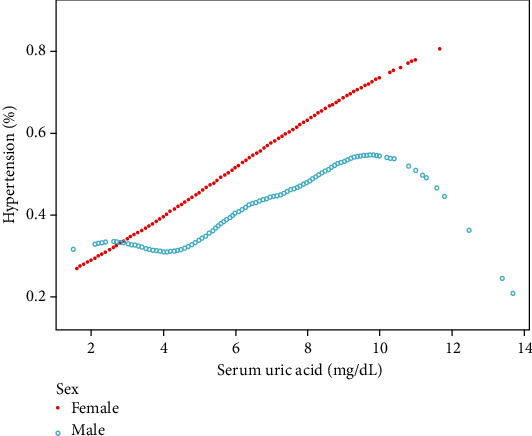
Association between sUA and HTN stratified by sex. Age, race, income-poverty ratio, physical activity, smoking behavior, alcohol consumption, diabetes mellitus status, body mass index, total cholesterol, serum homocysteine, urine creatinine, blood urea nitrogen, and total calcium were adjusted.

**Figure 4:**
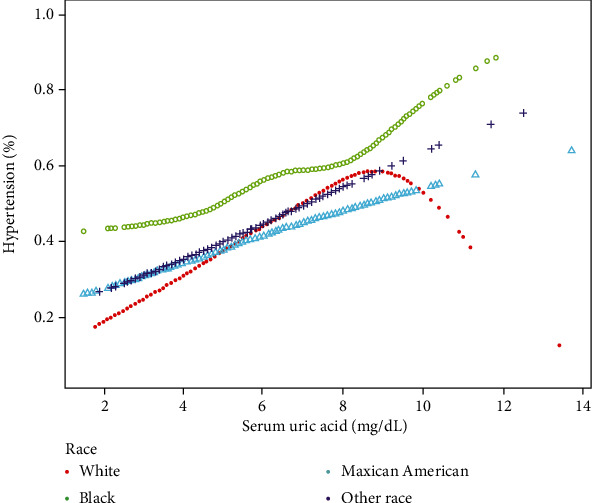
Association between sUA and HTN, stratified by race. Age, sex, income-poverty ratio, physical activity, smoking behavior, alcohol consumption, diabetes mellitus status, body mass index, total cholesterol, serum homocysteine, urine creatinine, blood urea nitrogen, and total calcium were adjusted.

For subgroup analysis (Table 3), a significant association between the sUA level and incidence of HTN was found in all female except those of other races (*P* for trend = 0.1154). In the male population, the sUA level was significantly associated with the incidence of HTN (*P* for trend > 0.05), except for Blacks and other race subjects. We tried to use generalized additive models and smooth curve fittings to identify the nonlinear relationship stratified by sex and race. As shown in Figure 2, there was an overall linear relationship between sUA and HTN incidence but subgroup analysis showed a nonlinear relationship between sUA and the HTN incidence in male and Whites (Figures 3 and 4). The incidence of HTN did not increased with sUA up to the turning point in male (turning point: sUA 4.1 mg/dL) (Table 4). Likewise, there were turning points in Whites (turning point: sUA 7.9 mg/dL) (Table 4). Taken together, the association between sUA and the incidence of HTN in male and Whites followed an inverted U-shaped curve.

## 4. Discussion

Epidemiologic data suggested an association between sUA and HTN. However, due to the complex factors associated with sUA and HTN, there were many different views on this issue and controversial results had been reported in this limited body of evidence. Thus, we aimed to investigate whether sUA was independently associated with the incidence of HTN, using a large and nationally representative sample of middle-aged and elderly adults in the US. Study findings showed that the incidence of HTN was statistically significantly higher with the increasing baseline levels of sUA, indicating that the higher baseline sUA level is an independent risk factor for HTN. However, the association between the sUA level and the incident of HTN followed a U-shaped curve for male (turning point: sUA 4.1 mg/dL) and Whites (turning point: sUA 7.9 mg/dL).

Previous studies have found an association between sUA and HTN. A cohort study performed in the US showed a dose-dependent increase in the relative risk of HTN with increasing quartiles of sUA [18]. A meta-analysis enrolled 18 prospective studies with a total of 55607 participants concluded that a high level of sUA was an independent risk factor for HTN, and the risk ratio for incident HTN was 1.13 with each increase of 1 mg/dL in the sUA level after adjusting for potential confounding factors, which was similar to that in our study [19]. Mechanisms underlying the relationship between sUA and HTN include a reduction in endothelial nitric oxide, the activation of the renin-angiotensin system, and renal microvascular disease caused by smooth muscle cell proliferation, inflammation, and local renin-angiotensin system activation [8]. However, another observational study and the Mendelian randomized studies did not support a causal association between sUA and HTN [12–14]. These conflicting conclusions may be attributed to differences in demographic characteristics, study design, study size, controlling for confounding factors, etc.

The first interesting finding was that the relationship between sUA and the incidence of HTN in male followed an inverted U-shaped curve. For male, the incident of HTN did not increase with increasing sUA until the turning point (4.1 mg/dL). A cross-sectional study of 85286 Japanese workers found similar results [20]. A significant relationship was found between sUA and HTN when sUA was ≥5.3 mg/dL in male. It was observed that there was a different relationship between sUA and HNT in people of different genders [19]. Previous studies [21] have shown that men have higher circulating levels of sUA than women. This gender difference may be explained as a result of the inhibitory effect of estradiol on sUA, as estradiol inhibits the isolated urate-producing enzyme and also decreases circulating uric acid in a pharmacological manner [22, 23]. Moreover, sex hormones have a potential impact on the relationship between sUA and HTN, as sUA has been found to be associated with the internal carotid artery resistance index and pulse wave velocity in female, but this phenomenon has not been observed in male [24, 25]. Thus, to understand the mechanisms underlying sex differences, further studies on the role of sex hormones are needed. Gender differences should also be taken into account in the prevention of hyperuricemic complications [4, 26].

The second interesting finding was that the relationship between sUA and the incidence of HTN in Whites followed an inverted U-shaped curve. The incident of HTN decreased when sUA levels reached 7.9 mg/dL. So far, evidence linking sUA and HTN in different races was very limited. Although there were studies on the association between sUA and HTN in Japan and China, however, these studies were conducted separately for their countries, where the races were all Yellows and ethnic factors were not taken into account at the study design stage, which made it impossible to compare the association between sUA and HTN among different races. To our knowledge, this was the first study to report the association between sUA and HTN in different races in US adults. Although the association between sUA and HTN in Whites following a U-shaped curve was an interesting finding, this phenomenon cannot be explained due to the lack of existing evidence. Thus, future prospective studies with large samples for different races are needed for further validation. Polymorphisms in the regulator of G-protein signaling 2 gene have been reported to be associated with HTN in Blacks but not in Whites [27].

Therefore, we speculated that genetic differences may be a potential explanation for the presence of a U-shaped curve in Whites, which was different from other races. However, further studies on the role of genes are needed to understand the emphasized mechanism of ethnic differences.

### 4.1. Limitations

In this study, the representative samples of the multiracial population were included to better generalize of the US population; the large sample size enables us to conduct further subgroup analyses for sensitivity test and to adjust many potential confounding factors. However, limitations must also be acknowledged. First, due to the cross-sectional design of this study, the causal relationship between sUA and HTN cannot be elucidated. Second, the diagnosis of HTN was based on the patients' self-report, which may lead to the risk of bias. Third, participants with cancer were excluded because these special populations have a great influence on sUA and HTN. Thus, the conclusions of this study cannot be applied to these patients. Fourth, the bias caused by other potential confounding factors that did not adjust in this study is not excluded.

## 5. Conclusion

In conclusion, this cross-sectional study suggested that the sUA level positively correlated with the incident of HTN, in middle-aged and elderly adults. However, the association the between sUA level and the incident of HTN in males and Whites followed a U-shaped curve.

## Figures and Tables

**Table 1:** Characteristics of participants.

Serum uric acid	Total	Q1	Q2	Q3	Q4	*P* value
Age	59.56 ± 13.05	56.66 ± 12.66	59.63 ± 12.75	60.31 ± 13.04	61.15 ± 13.25	<0.0001
Sex (%)						<0.0001
Female	50.57	78.88	59.93	39.12	30.15	
Male	40.43	21.12	40.07	60.88	69.85	
Race (%)						<0.0001
Non-Hispanic White	50.16	46.29	49.68	51.78	52.19	
Non-Hispanic Black	19.24	17.14	15.37	20.38	23.29	
Mexican American	23.64	29.57	27.23	21.12	18.02	
Other race	6.96	7.00	7.71	6.71	6.50	
Educational level (%)						0.4106
Less than high school	35.67	33.57	36.56	36.67	35.63	
High school	22.30	21.83	22.32	21.96	23.01	
College graduate or above	42.03	44.60	41.12	41.37	41.36	
Body mass index (kg/m^2^)	28.92 ± 6.05	26.76 ± 5.40	28.37 ± 5.86	29.48 ± 6.21	30.63 ± 5.94	<0.0001
Income poverty ratio	2.73 ± 1.53	2.69 ± 1.55	2.71 ± 1.52	2.74 ± 1.53	2.79 ± 1.52	0.2925
Any hypertension (%)						<0.0001
No	57.19	68.57	60.89	56.58	45.02	
Yes	42.81	31.43	39.11	43.42	54.98	
Diabetes mellitus status (%)						<0.0001
No	82.54	84.12	84.28	83.77	78.44	
Yes	17.46	15.88	15.72	16.23	21.56	
Physical activity (100%)						0.0054
0	27.32	27.31	26.74	27.05	28.37	
1	27.50	26.05	29.81	28.21	25.39	
2	17.81	20.58	15.19	17.38	18.54	
3	27.37	26.06	28.26	27.37	27.69	
Smoking behavior (%)						<0.0001
No	48.32	57.60	49.58	47.01	40.78	
Yes	51.68	42.40	50.42	52.99	59.22	
Alcohol consumption (%)						<0.0001
No	73.69	80.85	74.91	74.08	66.24	
Yes	26.31	19.15	25.09	25.92	33.76	
Total cholesterol (mg/dL)	210.16 ± 41.25	209.02 ± 39.64	211.21 ± 41.11	208.49 ± 40.60	211.85 ± 43.21	0.0591
Serum Hcy (*μ*mol/L)	9.75 ± 6.09	8.50 ± 7.26	8.96 ± 4.55	9.83 ± 3.89	11.43 ± 7.49	<0.0001
Urine creatinine (*μ*mol/L)	10650.25 ± 6824.07	8936.80 ± 6387.75	9917.44 ± 6463.99	11454.07 ± 6570.79	11921.86 ± 7339.25	<0.0001
Blood urea nitrogen (mg/dL)	14.93 ± 6.31	13.05 ± 4.61	13.86 ± 4.77	15.06 ± 5.82	17.33 ± 8.17	<0.0001
Total calcium (mg/dL)	9.44 ± 0.40	9.36 ± 0.39	9.45 ± 0.41	9.48 ± 0.40	9.47 ± 0.40	<0.0001

Mean ± SD for continuous variables: *P* value was calculated by the weighted linear regression model. % for categorical variables: *P* value was calculated by the weighted chi-square test.

**Table 2:** Association between sUA and HTN.

	Model 1, OR (95% CI)	Model 2, OR (95% CI)	Model 3, OR (95% CI)
Serum uric acid	1.32 (1.27, 1.37)	1.36 (1.31, 1.42)	1.22 (1.17, 1.28)
Serum uric acid categories			
Q1	Reference	Reference	Reference
Q2	1.46 (1.25, 1.70)	1.48 (1.26, 1.73)	1.25 (1.06, 1.47)
Q3	1.85 (1.60, 2.15)	2.05 (1.75, 2.40)	1.53 (1.29, 1.82)
Q4	2.84 (2.45, 3.29)	3.22 (2.72, 3.80)	2.11 (1.76, 2.54)
Stratified by sex			
Female	1.63 (1.53, 1.73)	1.49 (1.40, 1.58)	1.30 (1.21, 1.39)
Male	1.28 (1.21, 1.35)	1.26 (1.19, 1.33)	1.17 (1.10, 1.24)
Stratified by race			
Non-Hispanic White	1.40 (1.33, 1.48)	1.48 (1.39, 1.57)	1.29 (1.21, 1.38)
Non-Hispanic Black	1.26 (1.17, 1.37)	1.31 (1.20, 1.43)	1.18 (1.07, 1.31)
Mexican American	1.18 (1.09, 1.27)	1.22 (1.12, 1.33)	1.18 (1.07, 1.30)
Other race	1.24 (1.09, 1.41)	1.27 (1.11, 1.47)	1.14 (0.97, 1.33)

Model 1: no covariates were adjusted; model 2: age, sex, and race were adjusted; model 3: age, sex, race, income-poverty ratio, physical activity, smoking behavior, alcohol consumption, diabetes mellitus status, body mass index, total cholesterol, serum homocysteine, urine creatinine, blood urea nitrogen, and total calcium were adjusted.

**Table 3:** Subgroup analysis stratified by race and sex.

Quartiles of serum uric acid	Incidence of HTN OR (95% CI)			
	Whites	Blacks	Mexican Americans	Other races
Female				
Lowest quartiles	Reference	Reference	Reference	Reference
2nd	1.38 (1.04, 1.85)	1.25 (0.78, 1.99)	1.58 (1.07, 2.33)	0.85 (0.41, 1.76)
3rd	1.67 (1.21, 2.31)	1.56 (0.94, 2.61) 0.0864	1.64 (1.02, 2.64) 0.0417	1.71 (0.74, 3.97) 0.2090
4th	2.48 (1.68, 3.67)	2.04 (1.14, 3.63)	2.14 (1.13, 4.05)	1.89 (0.75, 4.79)
*P* for trend	<0.001	0.0114	0.0074	0.1154
Male				
Lowest quartiles	Reference	Reference	Reference	Reference
2nd	1.59 (0.96, 2.65)	0.54 (0.24, 1.18)	0.99 (0.54, 1.79)	0.36 (0.10, 1.34)
3rd	1.65 (1.02, 2.67)	1.03 (0.53, 2.03)	1.56 (0.88, 2.75)	0.41 (0.11, 1.47)
4th	2.61 (1.60, 4.24)	1.18 (0.61, 2.28)	1.59 (0.87, 2.89)	0.69 (0.19, 2.48)
*P* for trend	<0.001	0.1407	0.0396	0.5788

Age, sex, race, income-poverty ratio, physical activity, smoking behavior, alcohol consumption, diabetes mellitus status, body mass index, total cholesterol, serum homocysteine, urine creatinine, blood urea nitrogen, and total calcium were adjusted.

**Table 4:** Threshold effect analysis.

Serum uric acid	Adjusted OR (95% CI), *P* value
Male	
Serum uric acid < 4.1 (mg/dL)	0.79 (0.47, 1.31) 0.3574
Serum uric acid > 4.1 (mg/dL)	1.19 (1.11, 1.27) <0.0001
White	
Serum uric acid < 7.9 (mg/dL)	1.34 (1.24, 1.45) <0.0001
Serum uric acid > 7.9 (mg/dL)	0.85 (0.61, 1.18) 0.3195

Age, sex, race, income-poverty ratio, physical activity, smoking behavior, alcohol consumption, diabetes mellitus status, body mass index, total cholesterol, serum homocysteine, urine creatinine, blood urea nitrogen, and total calcium were adjusted.

## Data Availability

The survey data are publicly available on the Internet for data users and researchers throughout the world http://www.cdc.gov/nchs/nhanes/.

## Abbreviations

sUA: Serum uric acid
HTN: Hypertension
NHANES: National Health and Nutrition Examination Survey
US: United States
BMI: Body mass index
Hcy: Homocysteine
OR: Odds ratio.
